# Home care clients: a research protocol for studying their pathways

**DOI:** 10.1186/s12913-020-05363-7

**Published:** 2020-06-12

**Authors:** Janice M. Keefe, Laura Funk, Lucy Knight, Michelle Lobchuk, Marilyn Macdonald, Lori Mitchell, Julie Rempel, Grace Warner, Susan Stevens

**Affiliations:** 1 Department of Family Studies and Gerontology and Nova Scotia Centre on Aging, Mount Saint Vincent University, Halifax, Nova Scotia B3M 2J6 Canada; 2 Department of Sociology, University of Manitoba, Winnipeg, Manitoba Canada; 3 Nova Scotia Centre on Aging, Mount Saint Vincent University, Halifax, Nova Scotia Canada; 4 Helen Glass Centre for Nursing, University of Manitoba, Winnipeg, Manitoba Canada; 5 Faculty of Nursing, Dalhousie University, Halifax, Nova Scotia Canada; 6 Home Care Program, Winnipeg Regional Health Authority, Winnipeg, Manitoba Canada; 7 Helen Glass Centre for Nursing, University of Manitoba, Winnipeg, Manitoba Canada; 8 School of Occupational Therapy, Dalhousie University, Halifax, Nova Scotia Canada; 9 Continuing Care, Nova Scotia Health Authority, Halifax, Nova Scotia Canada

**Keywords:** Home care, Caregiving, Home care policy, Community health service needs, Aged, Longitudinal analyses, Mixed methods

## Abstract

**Background:**

Enhancing non-clinical home care supports and services for older adults to live well is a strategic priority in developed countries, including Canada. Underpinning these supports and services are structures of care that are reflected in home care policies, programs and practices within jurisdictions. These approaches to care exist at multiple levels and inform interactions, perceptions, and care assessment, planning and provision, ultimately shaping the supports that are delivered. Jurisdictional differences in approaches to care mean that pathways through home care systems may differ, depending on where one lives. The goal of this study is to understand how approaches to care shape the pathways of older adult home care clients with chronic and long term conditions in two Canadian health jurisdictions.

**Methods:**

This longitudinal mixed-methods study has three interrelated research streams informed by aspects of the socio-ecological framework. We will examine client pathways using a retrospective analysis of home care assessment data (Resident Assessment Instrument- Home Care) in two health authorities (Client/Service Data Stream). We will analyze interview data from older adult home care clients and a cluster of each client’s family or friend caregiver(s), home support worker(s), care/case coordinator(s) and potentially other professionals at up to three points over 18 months using a prospective qualitative comparative case study design (Constellation Data Stream). We will review home care policies relevant to both health authorities and interview key informants regarding the creation and implementation of policies (Policy Stream). Our study will apply an integrated knowledge translation (iKT) approach that engages knowledge users in research design, analysis and interpretation to facilitate relevancy of results.

**Discussion:**

Applying a mixed-method research design to understand approaches to care within and between two jurisdictions will contribute to the evidence base on older adult home care client pathways. Study results will identify how potential differences are experienced by clients and their families. An understanding of the policies will help to contextualize these findings. The iKT model will ensure that findings are useful for strategic planning and decision-making, and supporting changes in care practice.

## Background

Health and social care systems in Canada and other countries are struggling to meet the demand for care from older adults as a result of population aging [1]. In this context, home care and community supports are increasingly viewed as having an integral role in health care systems, and in facilitating ‘aging in place,’ across the globe. Older adults generally prefer to receive care in their own homes [2, 3]. Governments also increasingly look to home care as a sustainable approach with the potential to reduce public-payer costs by easing the pressure on waitlists for residential long term care, supporting earlier hospital discharge [3, 4] and promoting reablement. Evidence suggests that home care services can delay but not necessarily prevent institutionalization [5]. A recent report by the Canadian Institutes of Health Information found subpopulations of older adults in residential care who may not have required placement if appropriate community-based supports had been available [6]. This finding signals the need for ongoing attention to identifying unmet needs for supportive home care or service, and a nuanced understanding of service pathways and factors that influence service delivery in meeting client needs along the care path.

There is some concern, however, that increasing attention in home care to supporting short-term medical and complex care needs (e.g., post-acute, multi-morbidity) will lead to the erosion of preventive, non-medical and supportive functions of home care [7, 8]. This “quick fix” approach in home care moves away from attention to the prevention or maintenance of health and wellness to mitigate health and functional breakdown, and eventually institutionalization [9]. This can disadvantage older adult clients with chronic and long term care needs for supportive home care [10, 11], including personal care (e.g., bathing, shampooing, and dressing), housekeeping, meal preparation, transportation, and caregiver respite– otherwise known as non-clinical supportive home care, and the health system overall. Support with housework services, personal care and assistance, and meal preparation reduce the risk for institutional care [12–14]. Overall, evidence indicates that non-clinical supports can sustain functioning and independent living.

Although access to non-clinical supportive home care helps older adults to stay in their homes longer, certain factors may impact client trajectories or pathways within, and potentially out of, the home care system, with implications for clients, their family and friend caregivers, home support workers [15–17], and the system [8, 18]. For instance, clients’ use of home care services might maintain, decline or cease over time due to system-level approaches to care, that can restrict the availability of home care supports and services and client eligibility [19], creating barriers to access and utilization. Clients and families might also react to inconsistencies in care, high staff turnover [20] and perceptions of insufficient resources, inadequate and depersonalized care [21, 22] by reducing, ceasing or refusing their service use. Safety issues can also arise related to security, cleanliness and maintenance of homes, family and friend caregiver knowledge and skills [23], home support worker competencies, reassessments, and responsiveness in adapting care to changes in client and carer needs [18, 24–26]. When clients do not access appropriate home support services at critical points, and when family and friend caregivers are not adequately supported, service trajectories [27, 28] and outcomes are negatively impacted [28–30] and some can experience a potentially avoidable entry into long term residential care [31].

A few studies offer insights into family and friend caregiver experiences [32–35], including their interactions with the health and/or long term care system [36, 37], helping us to start to comprehend older adult care pathways over time. While medical care is most commonly delivered by professionals, family and friend caregivers provide many other necessary supports including transportation, help with domestic tasks and home maintenance duties, and emotional support [2, 38]. Recent research confirms that caregiver inability to continue is one predictor of increased likelihood of entering residential care [18], yet the recognition of the importance of supporting their role varies within the home care system [39, 40].

In Canada, where home care is not an insured service under the Canada Health Act, provinces, territories and geographic jurisdictions set their own parameters on the level and type of publicly funded home and community care that is provided. Due to differences in definitions, services and data collection and reporting measures, there is limited comparative information about home and community care across jurisdictions [3, 41]. Yet the availability of home and community based supports and services varies in different locations depending on jurisdictionally specific contextual factors, such as varying eligibility to receive care, public coverage of services, residency requirements, and access to services [42]. To date, no known studies have explored and compared how jurisdictional differences in approaches to care influence client and family and friend caregiver pathways through the home care system. By combining quantitative and qualitative research components, this study will generate evidence to address this gap and inform future home care policy and practice.

### Study goal and research questions

**FIG. 1:**
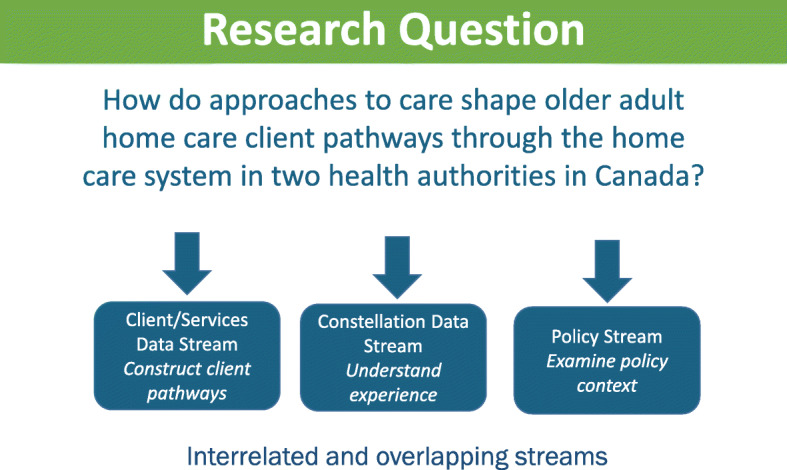
Research question and interrelated research streams

The overall goal of this study is to enhance understanding of home care client pathways of older adults with chronic and long term conditions through home care. Specifically, we will examine how approaches to care in two jurisdictions in Canada shape pathways through the home care system, from client and family and friend caregiver, provider and system level perspectives. Our overarching research question is: How do approaches to care shape older adult home care client pathways through the home care system in two health authorities in Canada? Three inter-related research streams (Client/Service Data Stream, Constellation Data Stream, Policy Stream) will address this research question (see fig. 1).

#### Approaches to care

Approaches to care, as defined in this study, encompass the philosophies and structures of care, as reflected and manifested in home care policies, programs and practices, within and across jurisdictions. Approaches to care inform client and care worker interactions, perceptions of family and friend caregivers, assessment and reassessment, care planning, care coordination, and care provision. Approaches to care at multiple levels (provincial, regional, office or agency) shape the kinds of supports and services that are either delivered publicly or purchased privately. These approaches influence who receives what level of services and for how long, the human resources and organizations needed to deliver and maintain standards of care, the expected contribution of family and friend caregivers, and the public and/or private costs associated with this care.

#### Scope

We will examine approaches to care by examining the home care pathways of older adult clients with chronic and long term conditions at two study sites in Canada. These sites are the Winnipeg Regional Health Authority (WRHA, one of five health authorities in the province of Manitoba) and the Nova Scotia Health Authority (NSHA, Nova Scotia’s single health authority). Nova Scotia (NS) and Manitoba (MB) have distinct geographic and cultural compositions, yet both are experiencing population aging and increased demand for home care services [43–46]. However, the home care systems have notably different staffing and delivery models. NS has a single health authority model in which agencies are funded by the Department of Health and Wellness (DHW) and contracted by the NSHA to deliver home care services to NSHA clients, which is distinct from the public provider model in MB in which health authority employees directly manage and, in most cases, deliver home care, through five health authorities. However, home care in both sites is embedded in a similar governance structure, whereby policy is set at the provincial health ministry level. Therefore, the case comparison of the sites will involve understanding relevant provincial policy and strategic direction.

## Methods

**FIG. 2:**
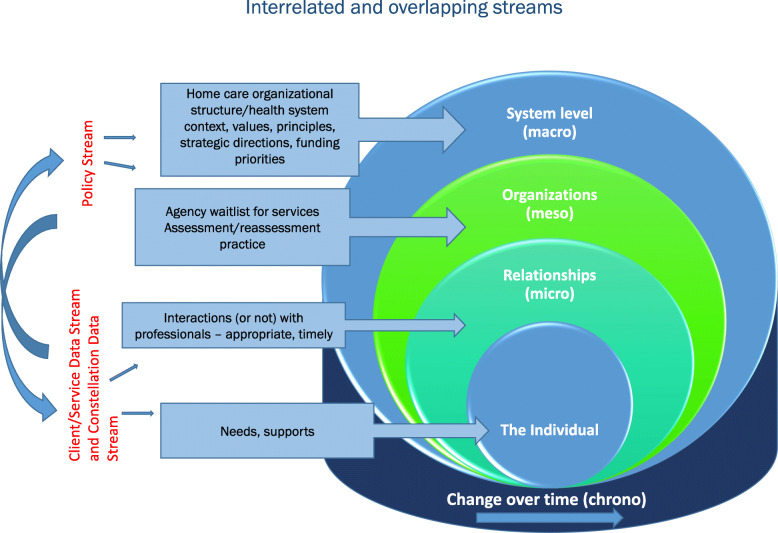
Guiding framework: Socio-ecological Model

The Home Care Pathways study is a longitudinal, two province, mixed-methods study involving a retrospective analysis of administrative data, alongside a prospective qualitative comparative case study of interview data and policy documents to identify when, where and how approaches to care influence home care client pathways (see figs. 1 and 2). We adopt a partnered Integrated Knowledge Translation (iKT) approach which is operationalized in the study’s structure ensuring partners are contributing to both knowledge discovery and application [47]. The study includes partners representing academic, decision-maker (provincial health ministry and health authority) perspectives and representatives from provider agencies on each stream-level working group. As well, the study’s management group, which includes academic and knowledge user members, share in the decisions regarding overall direction of the study.

### Guiding framework: a socio-ecological framework

The Socio-ecological Model is a systems framework, that informs this research design to help guide the examination of interlocking spheres of influence (e.g., chrono, macro, meso, micro systems) to illustrate how client pathways shape and are shaped by individual circumstances, social interactions and organizational and system-level approaches to care (see Figure 2) [48, 49].

Using a socio-ecological framework to inform all three research streams draws attention to the dynamic and reciprocal interplay of individual, family, community and organizational factors and the intersection of time throughout the analysis process. For example, our interpretation of individual circumstances as understood through the Client/Service Data Stream and Constellation Data Stream will be enhanced by recognizing that these circumstances are influenced by meso factors (e.g., a home care agency’s waitlist for services) which may influence resource allocation decisions (e.g., budget priorities at a funder level).

Research activities will occur over a three-year period, and are detailed below for each Stream.

### Research question # 1. What are the common pathways of older adult home care clients through the home care system? (client/service data stream)

The goal of the Client/Service Data Stream is to identify and examine person and health service-level factors related to an older client’s pathway – their clinical status and trajectory of care after admission to publicly funded home care. Our objectives are to: a) describe and understand the client population at admission to home care and over time; b) review quality of care, patterns of health service utilization after admission, and outcomes of home care clients; and c) compare and contrast clients’ clinical status and trajectory of home care between two provinces.

#### Methods

The Client/Service Data Stream involves a population-based retrospective cohort study that will use several MB and NS health data sources linked to a cohort of older adult home care clients. Clients admitted to home care will be followed for up to four years after their admission date to review change in clinical status and health system use over time. Client characteristics including health status, will be identified through the clinical assessment used by both the WRHA and NSHA, the interRAI assessment for Home Care (HC) (Resident Assessment Instrument RAI-HC). RAI-HC data from admission and follow up assessments will capture any change in client clinical status (improvement or decline) or maintenance in status over time. The RAI-HC initial assessment dates for the home care client cohort will be used to examine health service utilization in the following four years. Services examined will include amount and types of home care, emergency department visits, hospital stays, physician visits, and long term care admission.

#### Study cohort

The cohort will consist of all clients age 60 years and older at the time of admission to home care, who received non-clinical home care supports after admission. Older clients admitted to home care between January 1, 2011 to December 31, 2013, and who have at least one other assessment by the end of 2017, will form the study cohort. The cohort is estimated to be approximately 5500 individual home care clients in each study site, enabling techniques to be employed to detect relationships or difference.

#### Analysis

Data analysis for this study includes two phases. The first phase entails descriptive analysis to characterize the study cohort and changes in clinical status they experience over time based on the RAI-HC data. Home care assessment patterns and service allocation will be described along with other health service utilization. Retrospective analysis of clinical and health service utilization data will provide empirical data on actual interactions within the formal system and client patterns of utilization. The clients’ status at the end of the four-year follow-up will be reported – whether they remain in community, are admitted to long term care, or have some other disposition. The second phase of analysis will involve longitudinal analysis and multivariate modelling to estimate home care client trajectories. A number of variables will be examined for their potential effect on trajectories, such as changes in client health status, family caregiver involvement, and home care service levels, as well as home care assessment frequency and occurrence of other health service use.

The data linkage and analyses undertaken in this study will allow us to examine the various pathways of clients within the home care system and what factors are related to these pathways. Comparison of results between MB (WRHA) and NS (NSHA) will establish similarities and differences in client pathways between the two jurisdictions.

#### Challenges and mitigation strategies

Home care client data are protected by privacy legislation and accessible only by request to data custodians and ethics boards in each province. Timely data access and data linkage challenges will be mitigated by direct participation of health authorities and provincial health ministries. Contracting analysis of data at each site (WRHA and NSHA) to a single individual will promote replicability and consistency across the sites.

### Research question #2. How are service pathways shaped by (and in turn shape) the experiences of home care recipients, family and friend caregivers, home support workers and health care professionals? (constellation data stream)

The goal of the Constellation Data Stream is to explore individual experiences of clients, their family and friend caregivers (where available), and providers and coordinators of home care supports and services to understand how complex factors shape client service pathways in each study site, including identifying similarities and differences across urban and rural areas.

#### Methods

A prospective qualitative comparative case study design will be used, drawing on data from individual interviews conducted with members of 12 pre-defined care constellations (six in each study site). Interview questions will be initially retrospective to understand impetus for accessing home care service, and will include probes for specific experiences and examples of changes in care (as well as reasons for these changes). Care/Case coordinators and home support workers will be asked to comment on the client’s care plan but also more generally about policies shaping their work, as well as clients’ service use over time and decision making around service allocation and case management. Constellations will be followed over 18 months at three points in time every six months to observe potential changes in chronic care conditions of study participants and services provided. Findings will enhance understanding of contextual factors affecting client pathways (e.g., changes in cognition, resources), and the pathways preferred by clients.

#### Sample

Care constellations will be identified and theoretically sampled based on pathway typologies used in the Client/Service Data Stream: *improve, maintain, decline*. Each care constellation will include no less than three people, including a home care client aged 60 years or older residing in the community (with a Cognitive Performance Scale [CPS] score of 0–1), their care/case coordinator, and their home support worker. When available, that client’s family or friend caregiver will be included. Other professionals might be included depending on the care structure in respective jurisdictions; for instance, in NSHA, where care is delivered through contracted agencies, an agency supervisor will be included in the constellation.

It is estimated that between 42 and 54 individuals affiliated with 12 care constellations will be interviewed. This design maximizes the depth of data obtained for any particular care constellation and provides confidence that data saturation can be achieved. Specifically, the sample size will allow sufficient breadth and depth of data to identify patterns and build theory [50, 51].

#### Analysis

Transcribed interview data will be analyzed through complementary cross-sectional (thematic) [52] and temporal analyses – the latter to ascertain changes in experience of pathways over time [53]. Moreover, these cross-sectional and temporal analyses will attend to both within-constellation and between-constellation variation. This case study comparison approach [54] will be informed by the socio-ecological framework and findings from the policy review (Policy Stream described below).

#### Challenges and mitigation strategies

Team members from the two study sites will inform and support recruitment. Client recruitment will draw on expertise and experience of case/care coordinators to identify clients who fit the study criteria. In Nova Scotia, agreement will be obtained from four agencies to support their staff to participate. Strategies to address potential client attrition include recruiting clients at Time 1 with CPS scores of 0–1, and conducting exit interviews with other members of a constellation if a client dies. Team members have experience in the ethical engagement of research participants who may have cognitive impairment (e.g., recruitment, informed consent).

### Research question #3. How do policy contexts inform approaches to care and shape client pathways? (policy stream)

The goal of the Policy Stream is to contribute to understanding how the structure of home care in the two study sites can shape client pathways, through an analysis of the policy context of home care programs in NS and MB.

#### Methods

A policy document analysis [55, 56] and comparative case study design (where the province/jurisdiction is the case) will be used to review key policy documents that guide or structure how care is delivered. Key informant interviews will be conducted in each province to contextualize this analysis, which will also be informed by emerging findings or policy-related questions arising from the Client/Service Data Stream and Constellation Data Stream. Interview questions will focus on the intent/goal of the home care program, its values, and challenges and opportunities. Knowledge user team members will assist in the identification of relevant documents and key informants.

#### Sample

Documentation will include policies, guidelines, manuals, and other materials (including strategic documentation and website content) used by the provincial health ministries and health authorities (NSHA and WRHA) to guide home care. Key informant interviews will be conducted with approximately 10 senior decision-makers in each province, at health department, health authority, and agency (in NS) level to provide further insight into the development, operationalization and interpretation of the policies. This number of key informant participants is considered sufficient, given that the study has only two jurisdictions and the role of key informant interviews is to provide a secondary source of information to validate and/or enhance understanding and interpretation of information collected from the policy document analysis.

#### Analysis

A content analysis will be undertaken to describe the home care policy context in each jurisdiction (e.g., legislation, oversight, program delivery, caregiver support, scope of practice). Results from this analysis will provide the framework for better understanding the approach to care in each jurisdiction. These insights will enhance interpretation of other Streams’ findings into individual and family and friend caregiver experiences of home care, and service access and delivery considerations. Analysis of key informant interviews will contextualize findings from the streams and will help to identify policy actions and inactions that shape client pathways within and across the study sites, and the structural and contextual constraints and enablers in each jurisdiction.

#### Challenges and mitigation strategies

Home care services and approaches to care are multidimensional and complex [28, 44] emerging from the intersection of varying policies. Not all policies may be well known, or formalized, and those that are formalized may in fact have very little influence on operational aspects of home care service delivery. They may also be implemented in different and unintended ways. The iKT model and engaged knowledge users from health ministries and health authorities will guide our interpretation of the policy context and the implementation process, as will the information collected from key informants, and participants interviewed in the Constellation Data Stream.

### Ethics

Ethics approval for the Client/Service Data Stream and Constellation Data Stream was obtained using a two-site model, with oversight at Mount Saint Vincent University. For the Client/Service Data Stream, the WRHA study site ethics was approved by the University of Manitoba Health Research Ethics Board (HS22118) and the NSHA study site ethics by the Nova Scotia Health Authority Research Ethics Board. For the Constellation Data Stream, the MB study site ethics was approved by the University of Manitoba Psychology/Sociology Research Ethics Board (HS22462) and by the WRHA Research Access and Approval Committee (2019–003) and the NSHA study site ethics by the Nova Scotia Health Authority Research Ethics Board. For the Policy Stream, ethics approval for key informant interviews was obtained by the Mount Saint Vincent University Research Ethics Board. All site/stream level clearances received by ethics boards external to Mount Saint Vincent University obtained expedited review and clearance by the Mount Saint Vincent University Research Ethics Board, as the home institution for the Nominated Principal Applicant (NPA) and institution responsible for administering grant funds. Informed consent will be obtained from all interview participants (Constellation Data Stream and Policy Stream). For the Constellation Data Stream, consent will be ongoing and reviewed at each of the time points for the interview participants. While mild to moderate cognitive impairment of home care clients is not an eliminating factor, screening will occur as follows to ensure that cognitive impairment is minimal: care/case coordinators will be asked to select potential client participants with a CPS of 0–1; additional screening (in the form of questions confirming their comprehension of study information) will occur with clients at the time of informed consent [57].

## Discussion

Implementing a multi-dimensional study of this kind is complex. In a two-site model, in two jurisdictional contexts, timelines may diverge. With three streams, continued emphasis on the linkages between each stream’s research and the overall research question is important. However, the iKT model, together with a governance structure that includes high-level representation from each site, are strengths. This study’s findings stand to uniquely contribute to the evidence base on older adult home care client pathways over time. In addition to adding to what is known about home care in less studied jurisdictions of Canada (a gap identified by Johnson and colleagues) [58], the study will make the following contributions to scientific literature.

First, our study will take a longitudinal approach to comprehend care pathways through both a retrospective analysis of client pathways, and a prospective case study of care constellation member experiences over 18 months (while incorporating some retrospective data, for clients and families, on experiences over the last few years before Time 1). An understanding of home care policies in place during the time span covered by the service utilization data (2011–2017), as well as current policies will help to contextualize the research findings from the first two streams.

Second, our study outcomes will reflect unique cross-jurisdictional knowledge of approaches to care for older clients. For instance, many provincial publicly funded home and community-based programs and services claim to be organized by client-centered approaches to care [59]. However, it is not always clear how approaches to care are defined, translated into practice and policy, and how outcomes are evaluated. This makes it difficult to evaluate the influence of home supports and services on client pathways, and to assess the ability of the home care system to adapt and respond to changes in client care needs. Study results will identify potential differences between approaches to care as defined by the home care programs and how these are experienced by clients and their families. Comparing results from the two study sites will be a unique contribution that provides a nuanced understanding of the complexity of client pathways and provides an opportunity to analyze any commonalities or differences in experiences within the context of each jurisdiction’s distinct home care delivery model.

Third, the direct involvement of knowledge users from study conception to team composition and research design will facilitate timely access to client data (Client/Service Data Stream) and study research participants (Constellation Data Stream) and will guide our interpretation of the policy context (Policy Stream). Our knowledge users will aid in ensuring that findings are used to address knowledge gaps within the continuing care sector, and challenges can be better foreseen and addressed. Inclusion of knowledge users helps ensure that knowledge products will be relevant for provincial health ministries and regional health authorities, caregiver organizations and home care agencies.

Home care and non-clinical support services provided to older adults and family and friend caregivers in the community can vary highly across jurisdictions in Canada. By combining a novel longitudinal, mixed-methods design informed by the socio-ecological model and an integrated knowledge translation approach, this study will generate evidence to inform decision making and practice in home care specifically for two Canadian health authorities, and be of interest to other jurisdictions as well. This study uniquely incorporates clinical data and experimentation data from clients, family and friend caregivers, and those coordinating and providing care, about factors that shape care pathways as embedded in the policy landscapes of two provincial home care programs. We will produce knowledge products that identify, specify and characterize the types of care pathways, and delineate the approaches to care that shape them. Through our partnership of researchers and knowledge users, outcomes will be used to project utilization, inform strategic planning and decision-making, and support changes in care practice.

## Data Availability

Not applicable.

## Abbreviations

CPS: Cognitive Performance Scale
DHW: Department of Health and Wellness (Nova Scotia)
iKT: Integrated Knowledge Translation
MB: Manitoba
NS: Nova Scotia
NSHA: Nova Scotia Health Authority
NPA: Nominated Principal Applicant
RAI-HC: Resident Assessment Instrument – Home Care
WRHA: Winnipeg Regional Health Authority

## References

[1] National Institute on Ageing. Enabling the future provision of long-term care in Canada. Toronto: National Institute on Ageing; 2019. p. 161. Available from: https://cnpea.ca/images/futureoflong-termcare_v7_final-09-09-2019.pdf.

[2] Sinha M, Bleakney A. Receiving care at home. Ottawa: Statistics Canada; 2014. p. 18. Catalogue no. 89–652-X – No.002. Available from: https://www150.statcan.gc.ca/n1/pub/89-652-x/89-652-x2014002-eng.htm.

[3] Canadian Healthcare Association. Home care in Canada: From the margins to the mainstream. Ottawa: Canadian Healthcare Association; 2009. p. 152. Available from: https://www.healthcarecan.ca/wp-content/themes/camyno/assets/document/PolicyDocs/2009/External/EN/HomeCareCanada_MarginsMainstream_EN.pdf.

[4] Health Council of Canada. Seniors in need, caregivers in distress: What are the home care priorities for seniors in Canada? Ottawa: Health Council of Canada; 2012. p. 68. https://healthcouncilcanada.ca/files/HCC_HomeCare_FA.pdf.

[5] Kane R, Lum T, Kane R, Homyak P, Parashuram S. Does home- and community- based care affect nursing home use? J Aging Soc Policy. 2013;25(2):146–60. 10.1080/08959420.2013.766069. 10.1080/08959420.2013.766069 23570508

[6] Canadian Institute for Health Information. Seniors in transition: exploring pathways across the care continuum. Ottawa: Canadian Institute for Health Information; 2017. p. 45. Available from: https://www.cihi.ca/sites/default/files/document/seniors-in-transition-report-2017-en.pdf.

[7] Chappell N. Population aging and the evolving needs of older Canadians: An overview of the policy challenges*.* Montreal: Institute for Research on Public Policy; 2011. 31 p. Study No. 21.

[8] Toews R. Future of home care services in Manitoba. Winnipeg: Minister of Health, Seniors and Active Living (MB); 2016. p. 99. Available from: https://www.gov.mb.ca/health/homecare/future_homecare.pdf.

[9] Martin-Matthews A, Sims-Gould J, Tong CE. Canada’s complex and fractionalized home care context: Perspectives of workers, elderly clients, family carers, and home care managers. Canadian Review of Social Policy. 2013;(68/69):55–74.

[10] Neysmith S, Westhues A, Wharf B (2013). Caring and aging: examining policy inequities. Canadian social policy: issues and perspectives.

[11] Weeks LE, Keefe J, MacDonald DJ. Factors predicting relocation among older adults. J Hous Elderly. 2012;26(4):355–71. 10.1080/02763893.2011.653099.

[12] Chen YM, Berkowitz B. Older adults’ home- and community-based care service use and residential transitions: a longitudinal study. BMC Geriatr. 2012;12(44):1–12. 10.1186/1471-2318-12-44. 10.1186/1471-2318-12-44 PMC3444350 22877416

[13] Chen YM, Adams Thompson E, Berkowitz B, Ward D. Factors and home- and community-based services (HCBS) that predict older adults’ residential transitions. J Serv Sci Manag. 2011;4(3):368–79. 10.4236/jssm.2011.43043.

[14] Xu H, Weiner M, Paul S, Thomas J, Craig, B, Roseman M. et al. Volume of home and community-based Medicaid waiver services and risk of hospital admissions. J Am Geriatr Soc. 2010;58(1):109–15. 10.1111/j.1532-5415.2009.02614.x. 10.1111/j.1532-5415.2009.02614.x 20002513

[15] Panagiotoglou D, Keefe J, Fancey P, Martin-Matthews A. Job satisfaction and the context of care work by home support workers: Insights from three Canadian jurisdictions. Can J Aging. 2017;(36)1:1–14. 10.1017/S0714980816000726. 10.1017/S0714980816000726 28049546

[16] Denton M, Zeytinoglu IU, Davis S (2002). Working in clients’ homes: the impact on the mental health and well-being of visiting home care workers. Home Health Care Serv Q.

[17] Sims-Gould J, Byrne K, Craven C, Martin-Matthews A, Keefe J. Why I became a home support worker: recruitment in the home health sector. Home Health Care Serv Q. 2010;29(4):171–94. 10.1080/01621424.2010.534047. 10.1080/01621424.2010.534047 21153997

[18] Doran DM, Hirdes JP, Blais R, Baker GR, Poss JW, Li X, McIsaac C (2013). Adverse events associated with hospitalization or detected through RAI-HC assessment among Canadian home care clients. Healthc Policy.

[19] Fancey P, Keefe J. Home to nursing homes: Understanding factors that impact the path seniors take. 2014. Report contracted by the Nova Scotia Department of Health and Wellness, Continuing Care Branch.

[20] Dill DM, Keefe J, McGrath DS. The influence of intrinsic and extrinsic job values on turnover intention among continuing care assistants in Nova Scotia. Home Health Care Serv Q. 2012;31(2):111–29. 10.1080/01621424.2012.681526. 10.1080/01621424.2012.681526 22656913

[21] Aronson J. Elderly people’s accounts of home care rationing: missing voices in long-term care policy debates. Aging & Society. 2002;22(4):399–418. 10.1017/S0144686X02008759. 10.1017/s0144686x02008759 15264341

[22] Funk LM, Stajduhar KI, Cloutier-Fisher D. Exploring family caregivers’ rationales for nonuse of formal home health services when caring for a dying family member. Home Health Care Manag Pract. 2011;23(5):318–28. 10.1177/1084822310384920.

[23] Urbanik C, Lobchuk M. Encouraging family caregivers to “step inside the patient’s shoes”. Home Healthc Nurse. 2009;27(4):213–8. 10.1097/01.NHH.0000349906.89989.d1. 10.1097/01.NHH.0000349906.89989.d1 19387287

[24] Lang A, Macdonald M, Storch J, Stevenson L, Mitchell L, Barber T, et al. Researching triads in home care: perceptions of safety from home care clients, their caregivers, and providers. Home Health Care Manag Pract. 2014;26(2):59–71. 10.1177/1084822313501077.

[25] Lang A, MacDonald M, Storch J, Elliott K, Stevenson L, Lacroix H, et al. Home care safety perspectives from clients, family members, caregivers and paid providers. Healthcare Q. 2009;12:97–101. 10.12927/hcq.2009.20720. 10.12927/hcq.2009.20720 19667785

[26] MacDonald M, Lang A, Storch J, Stevenson L, Barber T, Laboni K, et al. Examining markers of safety in homecare using the international classification for patient safety. BMC Health Serv Res. 2013;13:191–201. 10.1186/1472-6963-13-191. 10.1186/1472-6963-13-191 PMC3669614 23705841

[27] Turcotte M. Canadians with unmet home care needs. Ottawa: Statistics Canada; 2014. p. 14. Catalogue no. 75–006-X. Available from: https://www150.statcan.gc.ca/n1/en/pub/75-006-x/2014001/article/14042-eng.pdf?st=5JpmlbSf.

[28] Robison J, Shugrue N, Porter M, Fortinsky RH, Curry LA. Transition from home care to nursing home: unmet needs in a home- and community-based program for older adults. J Aging Soc Policy. 2012;24(3):251–70. 10.1080/08959420.2012.676315. 10.1080/08959420.2012.676315 22720886

[29] Canadian Institute for Health Information. Supporting informal caregivers – The heart of home care. Canadian Institute for Health Information; 2010. p. 22. Available from: https://secure.cihi.ca/free_products/Caregiver_Distress_AIB_2010_EN.pdf.

[30] Gaugler J, Kane R, Kane R, Newcomer R (2005). Unmet care needs and key outcomes in dementia. J Am Geriatr Soc.

[31] Sarma S, Hawley G, Basu K (2009). Transitions in living arrangements of Canadian seniors: findings from the NPHS longitudinal data. Soc Sci Med.

[32] Cloutier DS, Penning MJ. Janus at the crossroads: perspectives on long-term care trajectories for older women with dementia in a Canadian context. Gerontologist. 2017;57(1):68–81. 10.1093/geront/gnw158. 10.1093/geront/gnw158 PMC5241789 27852640

[33] Turpin L. Ward-Griffin C. The meaning of a positive client-nurse relationship for senior home care clients with chronic disease. Can J Aging. 2012;31(4):457–69. 10.1017/S0714980812000311. 10.1017/S0714980812000311 23101877

[34] Fraser K, Nissen C. Uncovering the meaning of home care using an arts-based and qualitative approach. Can J Aging. 2014;33(3):246–58. 10.1017/S0714980814000191. 10.1017/S0714980814000191 25110980

[35] St-Amant O, Hall J. Making decisions in home-based dementia care: why context matters. Can J Aging. 2012;31(4):423–34. 10.1017/S0714980812000396. 10.1017/S0714980812000396 23217659

[36] Gruneir A, Fung K, Fischer HD, Bronskill SE, Panjwani D, Bell CM, et al. Care setting and 30-day hospital readmissions among older adults: a population-based cohort study. CMAJ. 2018;24(190):E1124–33. 10.1503/cmaj.180290. . 10.1503/cmaj.180290 PMC6157496 30249758

[37] Penning MJ, Cloutier DS, Nuernberger K, MacDonald SWS, Taylor D. Long-term care trajectories in Canadian context: patterns and predictors of publicly funded care. J Gerontol B Psychol Sci Soc Sci. 2016;73(6):1077–87. 10.1093/geronb/gbw104. 10.1093/geronb/gbw104 27558402

[38] Sims-Gould J, Martin-Matthews A. We share the care: family caregivers' experiences of their older relative receiving home support services. Health Soc Care Community. 2010;8(4):415–23. 10.1111/j.1365-2524.2010.00913.x. 10.1111/j.1365-2524.2010.00913.x 20298503

[39] Guberman N, Keefe J, Fancey P, Barylak L. ‘Not another form!’: Lessons for implementing carer assessment in health and social service agencies. Health Soc Care Community. 2007;15(6):577–87. 10.1111/j.1365-2524.2007.00718.x. 10.1111/j.1365-2524.2007.00718.x 17956410

[40] Guberman N, Lavoie JP, Pepin J, Lauzon S, Montejo ME. Formal service practitioners’ views of family caregivers’ responsibilities and difficulties. Can J Aging. 2006;25(1):43–53. 10.1353/cja.2006.0024. 10.1353/cja.2006.0024 16770747

[41] Hermus G, Stonebridge C, Thériault L, Bounajm F. Home and community care in Canada: an economic footprint. The Conference Board of Canada. 2012;23:1–66. Available from http://www.conferenceboard.ca/temp/65572e9a-c2b1-470e-81b1-651b35a01d36/12-306_homeandcommunitycare_prt.pdf.

[42] Williams PA, Lum J, Deber R, Montgomery R, Kuluski K, Peckham A, Zhu L (2009). Aging at home: integrating community-based care for older persons. Healthcare Papers: New Models for the New Healthcare.

[43] Health Association Nova Scotia. Rising to the challenge: Responding to increasing demands in home care. Bedford (NS): Health Association Nova Scotia; 2014. p. 56. Available from https://www.healthassociation.ns.ca/res/base/documents/library/research%20and%20policy/6%20home%20support%20-%20responding%20to%20increased%20demands%20-%20%20final20july207%202014.pdf.

[44] Ricard N. Manitoba home care program. Winnipeg (MB): Office of the Auditor General; 2015. p. 49. Available from: http://www.oag.mb.ca/wp-content/uploads/2015/08/Manitoba-Home-Care-Program-Report-Web-Version.pdf.

[45] Mitchell L, Roos NP, Shapiro E. Patterns in home care use in Manitoba. Can J Aging. 2005;24(Suppl 1):59–68. 10.1353/cja.2005.0053. 10.1353/cja.2005.0053 16080138

[46] Manitoba Health. Advancing continuing care: a blueprint to support system change. Manitoba: Health, Seniors and Active Living; 2014. p. 12. Available from: https://www.gov.mb.ca/health/documents/blueprint.pdf.

[47] Canadian Institutes of Health Research. Guide to knowledge translation planning at CIHR: Integrated and end-of-grant approaches. Ottawa: Canadian Institutes of Health Research; 2015. p. 34. Available from http://www.cihr-irsc.gc.ca/e/45321.html.

[48] McLeroy KR, Bibeau D, Steckler A, Glanz K. An ecological perspective on health promotion programs. Health Educ Q. 1988;15(4):351–77. 10.1177/109019818801500401. 10.1177/109019818801500401 3068205

[49] Andrew MK, Keefe JM. Social vulnerability from a social ecology perspective: a cohort study of older adults from the National Population Health Survey of Canada. BMC Geriatr. 2014;14:90. 10.1186/1471-2318-14-90. 10.1186/1471-2318-14-90 PMC4144321 25129548

[50] Morse JM (2000). Determining sample size. Qual Health Res.

[51] Morse JM (1995). The significance of saturation. Qual Health Res.

[52] Braun V, Clark V. Using thematic analysis in psychology. Qual Res Psychol. 2006;3(2):77–101. 10.1191/1478088706qp063oa.

[53] Thomson R, Holland J. Hindsight, foresight and insight: the challenges of longitudinal qualitative research. Int J Soc Res Methodol. 2013;6(3):233–44. 10.1080/1364557032000091833.

[54] Flyvbjerg B. Five misunderstandings about case-study research. Qual Inq. 2016;12(2):219–45. 10.1177/1077800405284363.

[55] Yanow D. Conducting interpretive policy analysis. Thousand oaks. CA: SAGE.

[56] Cheung KK, Mirzael M, Leeder S. Health policy analysis: a tool to evaluate in policy documents the alignment between policy statements and intended outcomes. Aust Health Rev. 2010;34(4):405–13. 10.1071/AH09767. 10.1071/AH09767 21108900

[57] Palmer BW, Dunn LB, Appelbaum PS, Mudaliar S, Thai L, Henry R, et al. Assessment of capacity to consent to research among older persons with schizophrenia, Alzheimer disease, or diabetes mellitus: comparison of a 3-item questionnaire with a comprehensive standardized capacity instrument. Arch Gen Psychiatry. 2005;62(7):726–33. 10.1001/archpsyc.62.7.726. 10.1001/archpsyc.62.7.726 15997013

[58] Johnson S, Bacsu J, Abeykoon H, McIntosh T, Jeffrey B, Novik N. No place like home: a systematic review of home care for older adults in Canada. Can J Aging. 2018;37(4):400–19. 10.1017/S0714980818000375. 10.1017/S0714980818000375 30176954

[59] Veteran’s Affairs Canada [Internet]. Client-centred approach. Government of Canada; 2015. Available from: http://www.veterans.gc.ca/eng/services/transition/case-management/approach#implement.

